# Identifying stakeholder preferences for communicating impact from medical research: a mixed methods study

**DOI:** 10.1186/s12913-024-11664-y

**Published:** 2024-10-29

**Authors:** Katherine Pitrolino, Buddhini Samarasinghe, Andy Pringle, Ian Viney

**Affiliations:** 1 College of Science and Engineering, University of Derby, Derby, DE22 1GB UK; 2 Evaluation and Analysis Team, Medical Research Council, Swindon, SN2 1FL UK

**Keywords:** Impact case study, Research outcomes, Medical research, Communication, Medical research council.

## Abstract

**Background:**

Documentation of research outcomes using impact case studies (ICS) is increasingly required to demonstrate the wider societal benefits of research. However, there is limited evidence of the best way to communicate research outcomes using ICS, especially when highlighting research impact that is not part of a research assessment programme. This study aims, for the first time, to analyse expectations, and methods of communicating impact from medical research across a varied set of stakeholders relevant to the Medical Research Council (MRC).

**Methods:**

Impact narratives about outcomes and impact from MRC research were evaluated using an online survey and in depth semi-structured interviews. Participants were recruited from internal MRC databases and included early career and senior management academics as well as representatives from industry, healthcare, charities, and the government. Informed consent was gained prior to data collection and the study was approved by the university’s research ethics committee. Qualitative and quantitative analysis determined stakeholder preferences for ICS content, language and presentation as well as capturing themes and perspectives on the concept of research impact.

**Results:**

193 participants responded to the online survey exploring definitions of impact and methods of communicating medical research outcomes. The work uncovered expectations of improved health and wellbeing as well as knowledge generation via publications and citations. In depth interviews with sixteen participants demonstrated preferences for clear, easy to read content that focused on facts and evidence and avoided both academic and hyperbolic language. Emergent themes from this work revealed that ICS need to quickly capture imagination and grab attention, while the views and expectations are quite different to press releases and are audience specific.

**Conclusions:**

The content of ICS often focuses on non-academic impacts; however this work highlighted that evidence of academic impacts were outcomes highly valued by stakeholders relevant to the MRC. This work examined a new typology of ICS attributes, which emphasised that the language and presentation of impact narratives can influence the perception of research outcomes, providing useful information for individuals and organisations using ICS to showcase their research. It also shows that if ICS attempt to communicate challenges and issues around achieving impact from research, they may be more credible and useful to their intended audience.

**Supplementary Information:**

The online version contains supplementary material available at 10.1186/s12913-024-11664-y.

## Background

Documentation of research outcomes and impacts is increasingly required to demonstrate the wider societal benefits of research [1]. Evidence of impact from academia is used to justify value for money to a range of key stakeholders, including sponsors and members of local communities and to support engagement with the wider public. This evidence can also provide a means of continuous improvement for organisations wishing to maximise the potential benefit from knowledge and technology transfer [2]. Importantly, impact assessments within the UK, Australia, Hong Kong, and Poland [3, 4] determine the level of government funding for research. These assessments, for example, Research Excellence Framework (REF) in the UK, use a common approach [4] including the presentation of impact evidence as case studies. Case studies provide much greater scope to explain, in both quantitative and qualitative terms, the variety of research outcomes and relevance to the economy and society than narrow publication metrics [5].

Impact narratives used for assessments have strict criteria and are structured to improve consistency during peer-review [1]. However, the periodic nature of assessments, every seven years in the UK, and the requirement to only submit a selection of research case studies mean they cannot reflect the full breadth of impact achieved from all research or all researchers [6]. It is also worth noting that the motivation is almost always to highlight positive impact. As such, examples of negative findings and disbenefits are rarely highlighted [5]. In addition to research assessment exercises, evidence of the impact achieved across a range of beneficiaries and environments [6] is often required by sponsors on an ad hoc basis [7]. For example, impact case studies (ICS) are increasingly being used by publishers [8], funders [9, 10], charities [11], and government organisations [12], to demonstrate the benefits of research.

The Medical Research Council (MRC) is one of the constituent councils of UK Research and Innovation (UKRI) [13]. Within MRC, impact narratives are held in a central internal database [14]. These include web-based articles from MRC Institutes, Units and Centres, reports from internal and external evaluations, impact case study (ICS) examples from the Research Excellence Framework assessments (REF2014 and REF2021), news articles from universities web sites, and summaries of outcomes reported via the Researchfish^®^ service [14, 15]. Narratives are collated to demonstrate the added value of MRC investments in research [16].

The purpose of writing an ICS may vary significantly depending on the organisation. ICS may be written by funding organisations to highlight the outcomes of a particular funding strategy, mechanism, or research programme. They may also illustrate support for multidisciplinary work, international collaboration, or describe translational advances made in specific areas such as prevention, diagnosis, or treatment. They may be written by research performing organisations to highlight research strengths and expertise, provide greater attention for key publications, or explain the positive impacts of particular investment decisions. Previous evaluations using REF ICS have been undertaken, and particularly those that correlate to high assessment scores [1, 2]. However, no such evaluation has taken place on more varied examples of ICS that are used to highlight research impact on a less formal and more frequent basis.

Analysis of REF ICS has identified three distinct aspects to a case study: (1) Content, (2) Language, (3) Presentation [17]. We used this approach as a high-level framework to examine what stakeholders value in ICS that are not prepared according to the REF requirements. This is an area with limited published research and to the best of our knowledge this approach has not been attempted previously.

Our study asked the following questions:

1. What types of impacts are valued by MRC stakeholders?
2. What kind of content are MRC stakeholders looking for when they read an ICS?
3. Does the language and style of the ICS influence the stakeholders’ view of the case study?
4. Does the presentation of the ICS influence the stakeholders’ view of the case study?

## Methods

This study adopted a mixed-methods research design [18]. The project was divided into three stages to investigate the research questions: - (i) initial scoping stakeholder interviews to gather views on ICS; (ii) experimental semi-structured interviews where stakeholders were asked their preferences from a list of attributes and a selection of four example ICS; (iii) large scale online survey to gather stakeholder views on research impact, their expectations of impact from MRC research and current methods of communication.

### Data collection

#### Scoping interviews

The study began with a search of the literature to identify any relevant previous work. A small selection of stakeholders internal and external to United Kingdom Research and Innovation (UKRI) were then interviewed to build a picture of the structure of impact narratives and how they were used within UKRI.

Key attributes contained within a selection of ICS were initially identified by KP, and grouped as “content,” “language” or “presentation,” using the key areas determined previously [17]. These attributes were piloted to 4 internal and external stakeholders (2 female, 2 male) who refined and added to them to produce the final list seen in supplementary information, (appendix 1, Table 1A). There were 11 individual scoping interviews and one scoping interview with two participants; representatives (9 female, 4 male). The interviewees were based in MRC, UKRI corporate hub, BBSRC (Biotechnology and Biological Sciences Research Council), STFC (Science and Technology Research Council), EPSRC (Engineering and Physical Sciences Research Council), DSIT (Department for Science, Innovation and Technology), and a medical research charity.

To assure methodological rigour, once these key attributes were validated, four examples of ICS were chosen from an internal MRC database to form a sample pack. The sample pack focused on less formal and more frequently published forms of impact communication such as news stories and website articles, and did not contain ICS examples submitted to the REF. The selected case studies demonstrated how the key attributes could be used to create an impact narrative. Each case study used different attributes and so the content, language style, and presentation varied between samples. The samples were taken from published online sources but were edited to less than 650 words to ensure homogeneity in length. The sample pack and associated attributes are detailed in supplementary information, appendix 2, Table 2A and Table 2B.

#### Semi-structured interviews

The sample pack was sent to a further 16 MRC stakeholders (6 female, 10 male) via email and their views were captured during a follow up video interview which was recorded on Microsoft Teams with their informed consent. The participants were selected from a list of MRC stakeholders that had already expressed interest in participating in the study and had given their consent to be contacted and to participate in the research. The research project took place according to the procedure approved by University of Derby College of Science and Engineering Research Ethics Committee, ref: ETH2223-3159. The stakeholders included representatives from Academia (senior management (*n* = 3) and early career researchers (*n* = 2)), Government [2], Industry [3], Medical Research Charities [3], and the National Health Service (NHS) [3].

The semi-structured interviews took approximately 30 min and although they followed the same initial set of questions there was space to allow follow-up questions and participant led areas of discussion. The participants were asked to rank the four ICS in the sample pack in order of preference and provide reasons for their choices. They were then introduced to the ICS attributes and asked their views on whether they should be included in an impact narrative. The attributes were scored on a 5-point Likert scale ranging from 1 (strongly agree) to 5 (strongly disagree) [19, 20]. At the end of the interview participants were asked whether they would include any more attributes in an impact case study and if they would change their ranking of the four ICS.

#### Online survey

Alongside the qualitative interviews, a separate online Qualtrics survey was created and distributed to almost 1,000 MRC stakeholders using email distribution lists from MRC Stakeholder Engagement and MRC Training and Careers teams. The self-selecting online survey took between 5 and 10 min to complete and asked for definitions of research impact as well as views on the expectations of impact from the MRC and the best ways to communicate it. The survey was developed uniquely for this study and is detailed in the supplementary file 2. Expectations of impact were determined by asking participants to score from 1 to 10, (where 1 is not expected and 10 is fully expected), a list of non-academic impacts as defined by REF2021 [21]. In addition to the list of impacts defined by REF, an indicator of academic impact, “high number of publications and citations” was added, the full list of impacts is shown in supplementary information, appendix 3, Table 3A. Responses were removed due to incompletion which was determined by Qualtrics software as more than 37.5% of questions unanswered. The response rate to the survey was over 20%: 193 participants answered the survey, and 133 responses were classified as complete. Participants included representatives from academia, the healthcare and third sector, industry, and government.

### Data analysis

A mixed methods approach [18] utilising both qualitative and quantitative techniques was used to integrate the data from the interviews and questionnaire. The scoping interviews were used to guide the questions for the online questionnaire and semi-structured interviews. The semi-structured interviews were transcribed and analysed using the six step process for thematic analysis [22], initial emerging themes were coded and discussed within the research team before developing the final set of coded themes. The qualitative responses from the online questionnaire detailing descriptions of impact were analysed using ‘a priori’ themes from the UKRI statement of impact [23] and the data was transformed to allow multivariate quantitative analysis. Minitab statistical software version 20.1.2 was used to analyse the attribute data and expectations of impact data using one-way ANOVA, with Tukey post hoc testing for multiple comparisons, p values were set to *≤* 0.05. Excel was used to graphically illustrate the transformed qualitative data from the online questionnaire.

## Results

**Fig. 1:**
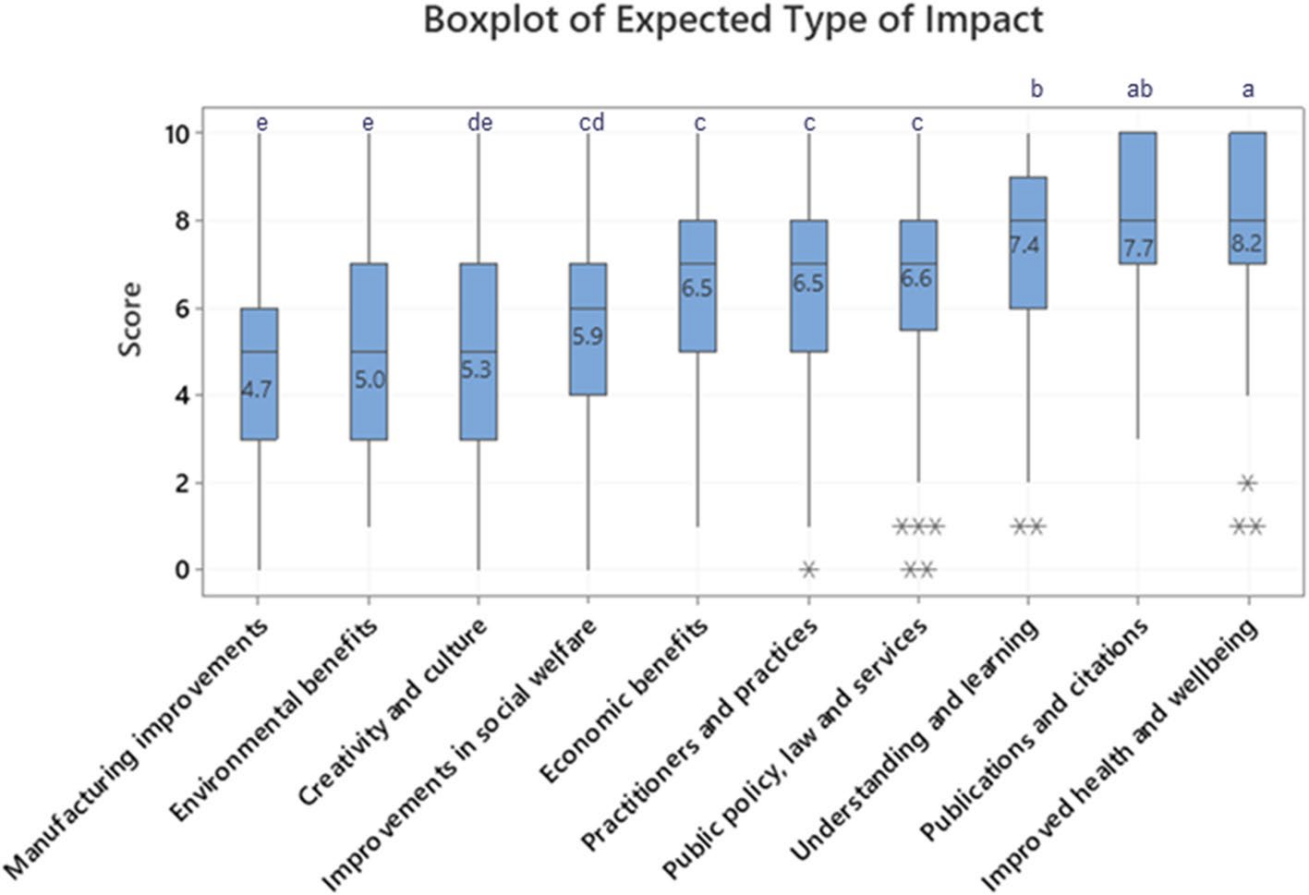
Boxplot of scores indicating MRC stakeholder’s expectations for different types of impact from MRC research (participants were asked to what extent they expected various types of impact, 1 = does not expect, 10 = fully expect that type of impact). Data was analysed using one-way ANOVA with Tukey’s post hoc testing for multiple comparisons. Means that do not share a letter are significantly different, *p* value ≤ 0.05

**Fig. 2:**
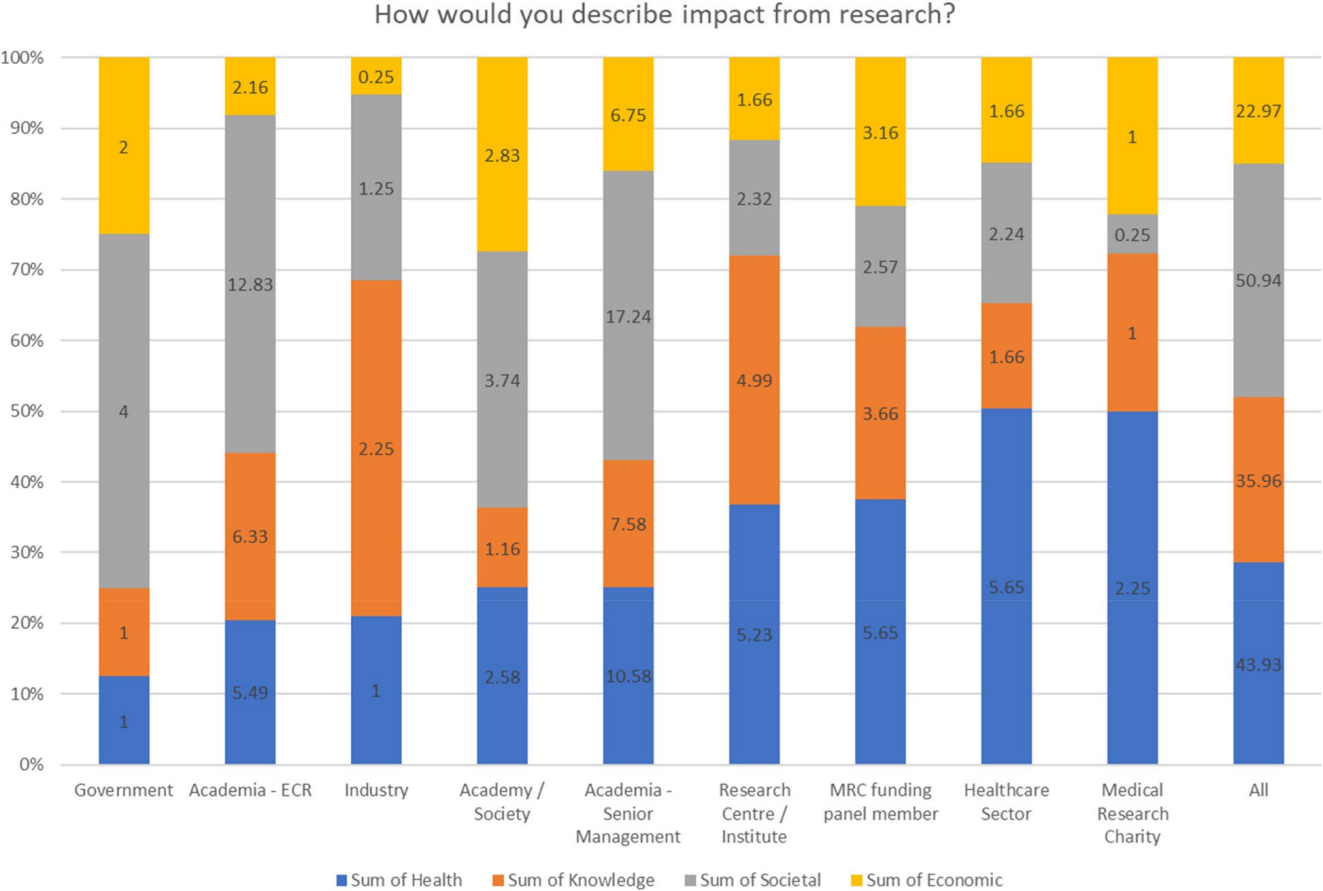
Stacked Bar Chart showing percentage of respondents mentioning types of impact aligned with UKRI’s statement of impact (this includes health, knowledge, societal, and economic impacts). The numbers of each respondent are displayed on the bar chart in each area. Where a stakeholder represented more than one role or sector, fractional counting was used to determine the percentage contribution

**Fig. 3:**
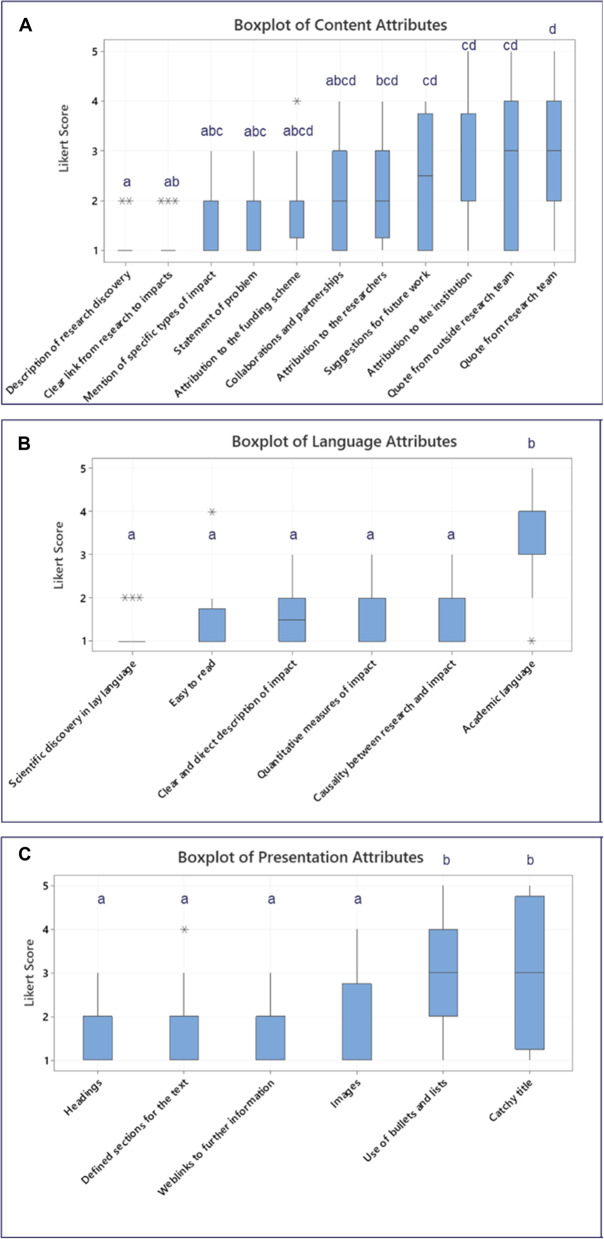
Boxplot and interval plots of Likert scores indicating MRC stakeholder preferences for impact case study content (**A**), language style (**B**) and presentation (**C**) (participants were asked whether an attribute should be included in an impact case study, 1 = strongly agree, 2 = agree, 3 = neither agree nor disagree, 4 = disagree, 5 = strongly disagree). Data was analysed using one-way ANOVA with Tukey’s post hoc testing for multiple comparisons. Means that do not share a letter are significantly different, p value ≤ 0.05

**Fig. 4:**
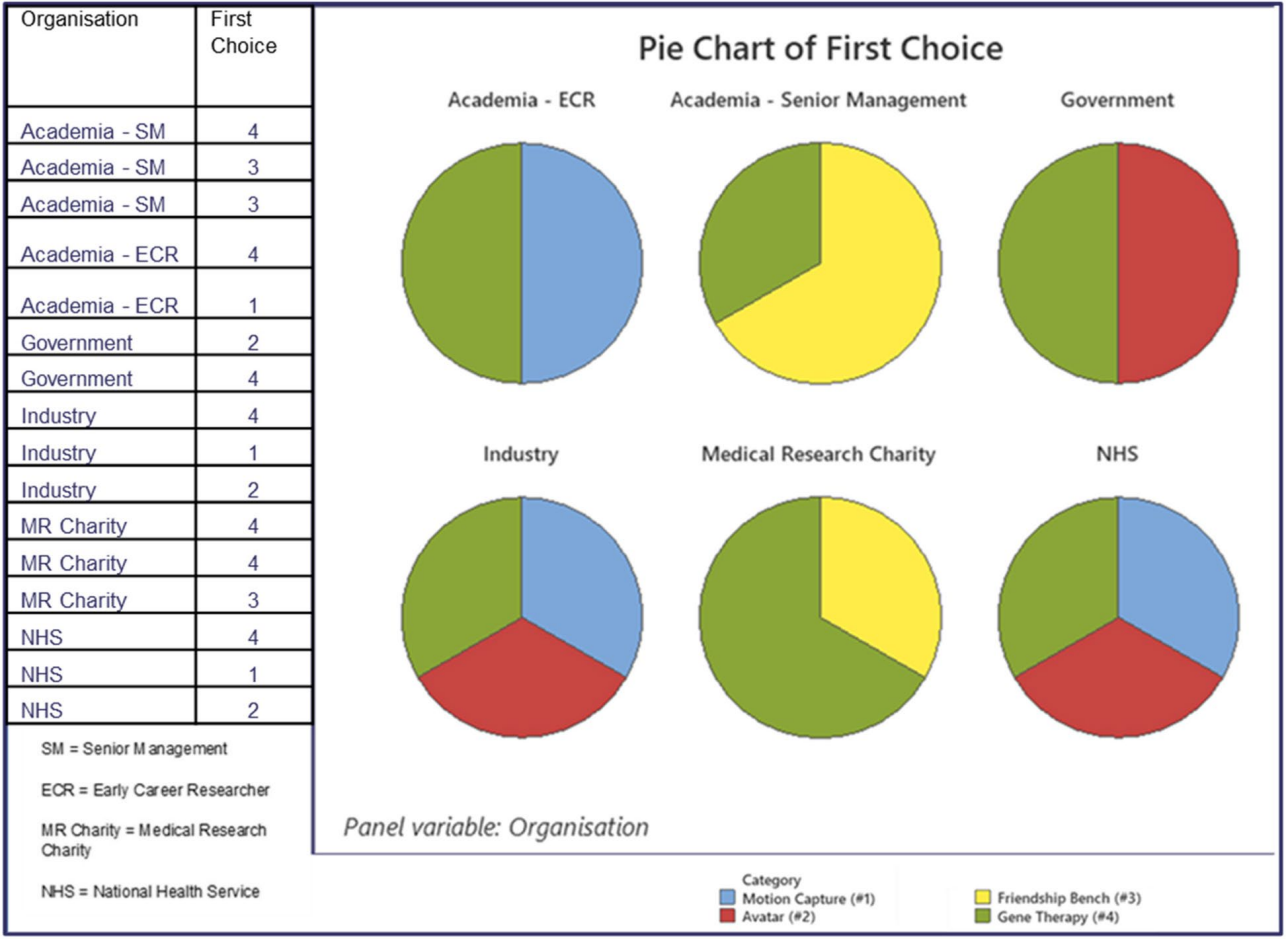
Pie Chart showing first choice preference for impact case studies from the sample pack by primary sector of MRC stakeholder

The following results show the outcomes from the semi-structured interviews and the online survey. Figure 1 shows the ratings for the types of impact expected by MRC stakeholders and Fig. 2 shows the responses from the open-ended question on descriptions of impact. Scoring of the content, language and presentation attributes, which suggest those most important to include in an ICS, is shown in Fig. 3 and additional attributes suggested by the participants are shown in the supplementary information, appendix 4, Table 4A. Preferences for the type of ICS from the sample pack are shown in Fig. 4. Key themes emerging from the interviews and the online survey are then discussed in detail [22].

### Expectations of impact from MRC stakeholders - online survey

To answer the research question “What types of impacts are valued by MRC stakeholders?” an online Qualtrics survey was circulated to MRC stakeholders via an email invitation. Participants were asked to rate the type of impact they expected to see from MRC funded research. Figure 1 shows the top three scoring types of impact were significantly different to the remaining types of impact. This indicates a focus on “improved health and wellbeing”, “high number of publications and citations” and “improvements in understanding, learning and participation” among MRC stakeholders. Government stakeholders scored “economic impacts” higher than average with a mean score of 8.3 for this impact compared to an overall average of 6.5.

An open-ended question asked participants to describe impact from research and the responses were analysed according to ‘a priori’ themes from UKRI’s statement of impact [23]. A word cloud showing stakeholders’ descriptions of research impact is shown in the supplementary information, Fig. 5A.

Each statement was analysed and categorised in terms of whether it referenced knowledge-based, economic, societal or health impacts. Around four fifths (79%) of stakeholders mentioned at least one of these types of impacts in their description. There were also descriptions of impact having a change on “policy and practice,” the “environment” and “developing people and careers” and so these were added to the “a priori” themes and it was noted 8% of respondents mentioned these in their answer.

Figure 2 shows the types of impacts mentioned from different stakeholders, with “all” depicting the sum totals for all the stakeholders. Where a stakeholder represented more than one role or sector, fractional counting was used to determine the percentage contribution for each type of impact. The figure shows all types of impact were mentioned by all types of stakeholders. The data is arranged in order of health impact with government sources mentioning this type of impact the least and stakeholders from medical research charities mentioning these the most. Knowledge impact was mentioned most frequently by participants from industry, while government sources tended to focus on societal and economic impacts. Societal and health impact were the most common combination of impacts as between 40 and 50% respondents mentioned these, over 30% included knowledge in their description and just over 20% mentioned economic factors in combination. It was noted that no participants gave descriptions of economic impacts in isolation.

### Scoring of attributes

During the semi-structured interviews, selected MRC stakeholders were asked to score the ICS attributes on a scale of 1–5 where 1 = strongly agree and 5 = strongly disagree on whether the attribute should be included in an ICS. Figure 3 shows the median and range Likert Score of content, language, and presentation attributes from the interviews. There were significant differences between *“quote from the research team”* and four other content attributes; *“description of the research discovery*,*” “clear link from research to impact*,” *“mention of specific types of impact” and “statement of the problem.”* This indicates that stakeholders would prefer to see those attributes in an ICS rather than *“quote from the research team*.” There were no significant differences between the scores of other content attributes.

There were significant differences between *“academic language”* and all other language attributes, indicating stakeholders preferred lay language to academic language in an ICS.

Figure 3A, B and C, show several content and presentation attributes divided opinion, with Likert scores ranging between 1 and 5, including *“attribution to the institution”*,* “quote from the research team”*,* “quote from outside the research team”*,* “use of bullets and lists”* and *“catchy title”*. More participants agreed there should be a “*quote from outside the research team”* (31%) than a *“quote from the research team”* itself (19%).

There were no language attributes with this range of variation between scores.

Figure 3A and B shows all participants (100%) agreed an ICS should include a “*description of the research discovery*”, a *“clear link from research to impacts”* and consist of the *“scientific discovery explained using lay language”*. A high proportion of stakeholders (94%) also agreed that the narrative should be *“easy to read”* and contain *“headings”*,* “images”* and *“weblinks for further information”* as shown in Fig. 3C.

Over four fifths of stakeholders (88%) agreed the case study should include *“statement of the problem with quantitative indicators”* and directly *“mention the specific type of impact”*. They felt there should be a *“clear and direct description of the actual impact”* and the text should demonstrate *“causality between the research and the impact”* using “*defined sections”* for the text.

There was disagreement among participants (Likert scores ranging between 1 and 5), about the extent to which the ICS should include specific attribution to the researchers involved in the work and/or attribution to the institution. However, more stakeholders agreed that the funding scheme should be mentioned (81%), than the researchers (62%) or institution (44%). Comments made during the interviews in relation to *“attribution to the funding scheme”* indicated that the funding scheme could give valuable information and help readers understand the significance of the ICS. They also felt the funding scheme had made a valuable contribution to the impact which should be acknowledged in the narrative. A presentation attribute that divided opinion was *“use of bullets and lists”* which were regarded as useful by some and off-putting by others, with some participants from industry commenting that “*the bullet points help you understand the key facts and messages*” while others from a medical research charity noted “*you lose the story when you’re listing things for people*”.

### Impact case study preferences

To test stakeholder preferences for attributes in the context of an ICS, participants were asked to read four different ICS provided in a sample pack and rate them in order of preference. The ICS, and their associated attributes are shown in the supplementary information file. The first choice of stakeholders is displayed in Fig. 4 and shows stakeholders from all organisations preferred ICS #4.

ICS Gene Therapy (#4) summarised 20 years of MRC discovery science and detailed a variety of impacts such as lives saved and spin out companies created. A single criticism was that it was not well written, with stakeholders commenting on the need for a clearer, more connected, and coherent narrative.

Figure 4 shows the remaining first choices were divided equally between the other ICS among the different stakeholders. It shows participants from academia did not choose Avatar (#2) as their first choice. This case study was written by a BBC news team and focused on the findings of a clinical trial that used motion capture technology to predict disease progression in people with movement disorders. ICS Friendship Bench (#3) detailed the implementation of a psychological intervention developed in Zimbabwe called the Friendship Bench. It was preferred by some stakeholders due to its concise nature and easy to understand impact. Stakeholders also appreciated that it described a cost-effective solution that had global application. Senior management stakeholders from academia and medical research charities liked this case study. Medical research charity stakeholders chose ICS #3 and #4 as their first choices and an analysis of their reasons indicated they preferred actual impacts rather than potential impacts or high-quality journal publications. This reason was also reflected in other stakeholder comments about ICS #4.

"*there is a clear statement about outcome*." MRC stakeholder from medical research charity about #4

"*like [the] fact it was a tangible impact, it had made a difference".* MRC stakeholder from NHS about #4

"*So, it’s not just an interesting finding, it’s an interesting finding that is being implemented in the real world and that continues to be expanded."* MRC stakeholder from industry about #4

Government stakeholders, however, were more interested in potential impacts than other stakeholders.

*“So a lot of things I’m looking for are statistics and evidence to illustrate the scale of the potential impact of the discovery……. [#2] was giving an illustration of the…. potential scale of impact." *MRC stakeholder from government about #2

Government stakeholders also liked the fact that ICS #2 was focused on an emerging area of research such as AI (Artificial Intelligence) and appreciated it *“captured some imagination”* with the Avatar reference and demonstrated “*the transition of technology between two very different spaces, so audio visual to medical”*. They also liked the economic impact mentioned in ICS #2 and had a particular economic focus in mind when they were reading the case studies, “*about trying to demonstrate value from a case study… [and perform a] cost benefit analysis.”*

When looking at the attributes mentioned above, 31% of stakeholders chose their preferred ICS because they liked the image, showing agreement with the attribute Likert scoring, where use of images scored highly. In general, language attributes were quoted as the main reasons for the choice of case study, this included attributes such as *“A clear and direct description of actual impact*,* (e.g. no use of word “potential”)”* which was quoted by 31% of the participants as influencing their choice.

Around 1/5th of the participants changed their preferred ICS after being presented with the typology of attributes, indicating knowledge of attributes of value can help determine ICS preference.

### Themes from semi-structured interviews

There was some opportunity during the interviews for participants to comment openly about their views on impact and impact narratives. Emergent thematic analysis was used to capture their comments and three separate themes emerged: (1) ICS need to quickly capture imagination and grab attention; (2) what makes a good ICS is audience specific; (3) views and expectations of ICS are quite different to a press release. The results from these three themes are detailed below.

1. ICS need to quickly capture imagination and grab attention.

It was clear from the semi-structured interviews that stakeholders are reading these ICS from a variety of sources which includes social media posts, news articles and website posts. They emphasised the limited time available to grab the reader’s attention and provide the motivation to read on, for example,

*“I like to know what I’m going to read. Do I have two minutes to read this in my busy lifestyle? I like to know exactly what I’m going to read so that I know that this is going to be of interest to me before I invest that time in reading and understanding it.” MRC Stakeholder from NHS*

"*In terms of pitching things to Treasury, it’s about trying to use things that are exciting or where something unexpected has happened."* MRC stakeholder from government

This perception of “time limited readers” created a need for ICS to be easy to read and concise, for example,

*“people nowadays, they have got such little time, they’re looking at this on their phone. They wanna know the key thing. They want that key takeaway. They need that summary of what it’s all about and then you can read on if you want.”* MRC stakeholder from medical research charity

*“Nobody ever reads it twice, they’ll read it once, they’ll read it over their cornflakes, it’ll take them 10 seconds”. *MRC stakeholder from industry

Several participants mentioned an informative image and clear title was critical in encouraging them to continue reading the ICS. For example;

*“it was in the title, “MRC funded discovery” so that gives me the context that this is MRC related, which didn’t happen in all of them, and it’s also*  *[a]* *“gene therapy cure”. So, you know, it’s straight away telling me what this is about”.* MRC Stakeholder from NHS

"*I will choose which ones to look into and potentially capture, often, depending on the titles within the newsletters that I read."* MRC Stakeholder from Government

"*I can get an idea of what will follow in the text just from [the image]."* MRC Stakeholder from Academia – ECR

2) What makes a good ICS is audience specific.

A variety of stakeholders from different sectors took part in the survey and semi-structured interviews. Several stakeholders commented that ICS preferences depended on the audience they were trying to reach. There was evidence that stakeholders were aware of a distinction between a narrative created for a public and a scientific or academic audience. Some stakeholders elaborated on this topic and detailed some content that would be more suitable for a particular audience, for example,

"..*being a scientist, I don’t need all of the glossy bits. I’m looking for the substance of what’s been discovered and what the advance is and how clear it is………if you’re writing this for the general public it may not be the same as the way I’m judging them……. I think everyone is different. This is hard, you know what, everything is bespoke, so I don’t think you can have one recipe fits all……I think knowing who it is designed for is really important."* MRC Stakeholder from Academia-Senior Management

"...*they’re quite different, which is quite difficult to compare. What one person thinks is impact is not necessarily the same as the definition as determined by REF."* MRC Stakeholder from NHS

3) Views and expectations of ICS are quite different to a press release.

There was a marked difference between what stakeholders thought about an ICS and a press release or topical news story. For example,

*“it does read like a press release, which doesn’t always work in terms of getting over the real value of something*.*"* MRC stakeholder from academia – senior management

"..*when I think about any impact study, I don’t want to read [it] in the news, it’s different from the impact study……the difference between…. research news and an impact case study is that there needs to be some academic message."* MRC stakeholder from academia – ECR

Concepts around including hyperbolic language and reactions to the use of a heading in one ICS highlighting “game changer” were regarded as negative attributes and could detract from the content and diminish the claims made. This was previously mentioned when assessing the preferences for an ICS from the sample pack and was due to the overuse of such words and the unrealistic idea that every new research breakthrough would deliver a paradigm shift in thinking. For example,

"*There’s lots in the medical world about breakthroughs and cures, so you have to use those words carefully and evidence that. People won’t read on if they think oh, here we go again."* MRC Stakeholder from Medical Research Charity

"*I know why they’re using game changer, but it’s such an overused phrase, I would discourage people from using it………it does read like a press release, which doesn’t always work in terms of getting over the real value of something."* MRC Stakeholder from Academia - Senior Management

Other differentiators to press releases involved the use of evidence to support the claims made. There was the view that press releases would exaggerate the finding, which is often a publication, whereas an ICS would focus on the demonstrated societal benefits of that research finding outside of academia. It was recognised however, that more could be done to support evidence synthesis and gather more data on the significance of the impact as well as challenges around the pathway to impact, for example:

"...*for this thing defined as an impact case study, I think it’s really important that it be evidence based……. I think the real value of something like the REF defined impact case study is that it has to be evidence based."* MRC Stakeholder from Academia – Senior Management.

*“impact case studies very often describe……a research breakthrough….[they] very rarely go on to attempt to quantify the scale of the impact."* MRC Stakeholder from Government.

"*I think it’s also important to talk about what couldn’t be achieved and what was learnt from that."* MRC Stakeholder from Academia – Senior Management.

### Themes from online survey

The open text in the descriptions of research impact from the online survey was also investigated using emergent thematic analysis and the following themes were identified: (1) impact promoting a positive change; (2) impact measures and assessment; (3) issues and challenges with impact.

1. Impact Promotes a Positive Change

Around three quarters of participants described impact in terms of promoting a change and within these over two thirds described a positive change, i.e. “Changing society or clinical practice for the better” MRC Stakeholder from Medical Research Charity. In describing change some respondents mentioned patients as the direct beneficiaries i.e. *“Changes in healthcare that improve outcomes for patients or society.”* MRC Stakeholder from Academia - Senior Management.

2) Impact Processes

17% of participants referenced the need to evidence impact and provide adequate measures, i.e. *“Measurable improvements to health and well-being.”* and *“Measurement of effects on policy development and application, governance and legislation, financial decisions, health care provision, other public services, etc.”* MRC Stakeholders from Academia - Senior Management.

The Research Excellence Framework was also mentioned, and it was pointed out that impact should not simply be defined by bibliometrics or REF definitions, i.e. impact is *“a powerful effect that something, especially something new such as a research finding has on a situation or person and definitely NOT the REF definition”* MRC Stakeholder from Medical Research Charity and *“Impact should not just be based on citations….[it] should be judged on the changes it makes to practice regardless of how big or small.”* MRC Stakeholder from Academia - ECR.

3) Impact Challenges

A few stakeholders described challenges around impact in their description, particularly around the timeline to impact, i.e. *“not all good research has an immediate impact, impact may take time”* MRC Stakeholder from Academia – ECR and *“Impact from research has many facets and perhaps an issue is trying to narrowly define it around short-term impact on human health.”* MRC Stakeholder from Academia - Senior Management.

Challenges with funding was also mentioned with some participants commenting substantial amounts of funding are required to notice real differences that can create impact i.e., *“It depends on the sector, but in my sector [psychology and mental health] you need a lot of funding and big samples to understand sociodemographic differences.”* MRC Stakeholder from Academia – ECR.

## Discussion

This paper set out to determine MRC stakeholder’s views of research impact and the ways it can be communicated. It focused on how the different types of stakeholders define impact from research and how case studies can be used to articulate the pathways to impact and describe the significance of the research findings. With that in mind, our paper answers the research questions and reports on the (i) expectations of impact from MRC Stakeholders, (ii) ICS content, language and presentation of value to MRC Stakeholders and (iii) ICS preferences specific to each stakeholder.

### Expectations of impact from MRC stakeholders

In this study, stakeholders were categorised by their work role and organisation. A key finding was agreement among different stakeholders about the type of impacts they expected from MRC research. The most expected types of impact were (1) improvements in health and wellbeing (2) a high number of citations and publications and (3) improvements in understanding and learning. This not only highlights MRC’s mission to improve human health but also an understanding that it primarily seeks to achieve this through supporting high quality discovery and translational research [10]. It reinforces the idea that academic impact and bibliometric measures are more important to MRC stakeholders than other types of impact, such as economic impacts.

### ICS content of value to MRC stakeholders

Content, language, and presentation aspects from ICS [17] were used to determine “attributes” and this study asked stakeholders to score these attributes in terms of preference. All participants agreed there should be a description of the research discovery and a clear link between the research and the resulting impact. This supports findings from a peer reviewed analysis of ICS from REF2014, where 97% of a sample of high scoring ICS clearly linked the underpinning research to the claimed impacts [17] contrasting with only 50% of low-scoring ICS.

There was disagreement between stakeholders whether there should be a mention of the researchers, institution, or funding scheme, with more participants agreeing that the funding scheme should be mentioned before the researchers or institution. More specifically and importantly, in the case of MRC funding, the funding scheme was also valued as a surrogate measure of the quality of the research. This should be of interest to sponsors looking for evidence of impact from specific funding mechanisms, and is an aspect that has not been explored in previous research around scientific news and press releases, where reference to the funding scheme was rarely found [24]. Attribution to other sources, however, such as the researchers or their institution, was not as valued by MRC stakeholders. It is suggested that this may be due to the perceived motivation of building a brand [25] and the alternative incentive to gain positive publicity.

More participants agreed there should be a quote from outside the research team to contextualise the research within the field and comment on the significance of the impact, rather than the research team themselves. Previous studies on news stories and press releases detailing research outcomes found quotes were omnipresent [26] and categorised them as part of “interest-raising measures” [26] which also include the use of “ground-breaking words”. This approach was commonly challenged by MRC stakeholders as it was thought to promote hyperbolic messaging and unnecessarily sensationalising research outcomes, which detracted from the key findings [27]. A US study in 2014 found differences between conflicting and confirming quotes given from outside the research team in specific scientific areas [28]. They found confirming quotes were more likely to be included in press releases where reliability of facts is valued [28]. Some MRC stakeholders agreed that quotes from beneficiaries or patient volunteers would be of value and could provide assurances to the significance and scale of the impact claimed in the narrative.

### ICS language and presentation of value to MRC stakeholders

There was agreement among stakeholders that ICS should use clear and direct lay language, with a structure that helped the reader by including headings and the use of images.

When asking MRC stakeholders their preferences from an ICS sample pack, readers often rated presentation and the type of language as more influential than the strength of the research finding or significance of the impact. This contrasts with the positive scoring given to content attributes related to the research finding or significance. It highlights a disconnect between what readers are looking for when presented with the attributes, and what engages them when presented with an ICS. This is often linked to limited time availability to read the ICS and may have relevance to peer review of ICS during a research assessment exercise.

Some stakeholders suggested images should be able to connect with the ICS story and not just used to draw attention more generally. This reflects the type of media used to communicate research outcomes as previous analysis on websites or web content found images are increasingly concerned with enticing the reader to click on the image rather than reflecting any meaningful information on the content [29]. Stakeholders mentioned that written narratives might not be the best way to disseminate research findings and alternate forms of media that could be deployed, such as video, were highlighted. This view also opens the possibility of research impact being communicated more effectively using other approaches such as citizen science, public engagement, and participatory methods [30, 31].

### Impact case study preferences

Although it was clear from the attribute scoring what aspects of an ICS were valued by our stakeholders, this did not always translate to their first choice ICS from the sample pack. We found evidence of an influential personal component, which may have triggered an emotional response to the narrative [32] reminding them of someone they knew or a research area of particular interest to them. This aspect is well recognised in public science communication and can lead to specific narrative framing [33, 34] relevant to a particular audience [31]. Using narratives to describe scientific research outcomes is prone to interpretation by audiences according to their “previous experiences, cultural context, and personal circumstances” [31]. This promotes audience segmentation based on advanced psychological personas to ensure the most effective reach. This may mean that the way ICS are framed is critical to how people perceive them and “turning to audience research to design messages that are personally relevant and meaningful to diverse publics” could be an effective communication strategy [33]. However, it is unclear if this approach is relevant to ICS.

Indeed, the themes from semi-structured interviews showed that perceptions of ICS are different from those of a press release. Previous work recognised that scientific press releases could exaggerate findings and omit information [24, 26, 35], even though “reliability of facts” was seen as the most important factor among a survey of science communicators [28]. Press releases that focused on a particular researcher or a university were thought to have a motive to build a brand [25]. These factors could lead to mistrust among audiences [36], an aspect also recognised in this work by MRC stakeholders assessing the sample ICS.

However, this study suggests there is an opportunity for impact narratives to gain more trust from an audience than typically expected from a press release by being objective, incorporating information supported by evidence and not using hyperbolic language. It was suggested that articulating the pathway to impact, including any issues and challenges as well as an assessment of “what worked and what didn’t,” could also build confidence in the narrative [34, 36]. There was some evidence of government audiences preferring ICS with unusual or unexpected findings. This has also been seen when assessing the reach of scientific new stories in the media where unusual or controversial findings tend to be more visible [37].

Many of the findings from this study have implications for researchers writing ICS and so a summary of recommendations is shown in Table 1.

**Table 1:** Recommendations and implications for researchers writing Impact Case studies

Recommendations
When selecting impacts to communicate progress, improvements in understanding and learning, as well as traditional measures of academic impact, are valued by the community alongside non-academic impact.
When writing impact narratives consideration of the intended audience is key and content should be tailored to these audiences.
Specific presentation and language attributes provide emotional aspects to the narrative and can determine preferences more than content attributes.
Care should be taken to include factual evidence to clearly describe the actual impact and what might be achieved in the future.
A narrative, explaining the pathway to impact and funding contributions, as well as highlighting the issues and challenges around generating impact is of value to the research community.

### Limitations and strengths

In assessing these findings, it is important to explore any limitations [38, 39]. We acknowledge that our interviewees may have given greater emphasis to attribution to the funding scheme, knowing that they were being interviewed as part of a study commissioned by the MRC. Although we were careful to state any views expressed would not affect future grant applications and made sure the interviewers were not part of the MRC’s grant decision making process, this is a limitation. This problem has been previously recognised in other work performed by a research funding organisation when gathering stakeholder views about impact narratives [36]. However, the conduct of the work as a secondment based within the MRC is suggested as a strength. Previous research on research studies have found it difficult to gain awareness within funding agencies, which have limited capacity to consider emerging findings and apply these to policy making [27, 40]. The secondment meant that the project was co-produced with UKRI employees directly connected with the development of ICSs, as well as access to mailing lists of stakeholders and curated sets of ICSs [41, 42]. We therefore have some confidence that the work has already made a positive difference to the way that the MRC evaluation team approaches ICS.

When evaluating the outcomes from the online survey, it was difficult to draw conclusions about participants from industry and government due to the small sample sizes. Larger sample sizes from these sectors are recommended to support and validate these findings. It was also apparent from the online survey that many MRC stakeholders were involved in various roles at several different organisations, for example, an academic could also be employed by the NHS and contribute to an industrial small and medium-sized enterprise (SME). This has implications for any inferences made on scoring of attributes as determined by role. When assessing the ICS preferences, different subject matter and language styles were used to determine stakeholders’ preferred impact. This was successful in drawing out preferred research areas and types of impact while also giving an insight into the preferred style and structure of the ICS. Indeed, two of the ICS used in the sample pack covered the same research outcome using different language and presentation, which was influential in extracting views on these attributes. However, sometimes it was difficult to determine the exact contribution of each of the three areas, i.e. content, presentation, and language on preference. It may be useful in the future to use ICS that were all similar in style and from the same source to ensure that participants focus on their preferred type of content.

A strength of the online survey was that it covered a large sample size for academia and therefore any inferences made for this sample sector could be seen as representative. It was also possible to add detail to the academia sample and gain information on whether the participant was an early career researcher, part of senior management and whether they had any experience of being an MRC Panel Member. The semi-structured interviews were designed so that there was an equal representation from all key sectors giving important insights into a wide range of views and preferences. By incorporating some participants from government and industry, an emerging trend was evident in which these stakeholders shared common views and values. This could be explored further if more substantial engagement of this group is possible.

The systematic approach used to test attributes of importance to a variety of MRC stakeholders is novel and provides quantitative evidence on preferences for impact case study content, language and presentation which previously was only assumed or suggested. This study provides an indication that preferences for ICS could be influenced by awareness of key attributes, which may be important for future communication of research outcomes. It could also have relevance for any future research assessment programme that includes the submission of impact narratives.

## Conclusions

This work shows expectations of impact from MRC stakeholders reflect views from UKRI strategy and recent national research assessment exercises [21, 23]. However, while assessment exercises focus on non-academic impact, these results would suggest that evidence of academic impacts is highly valued by stakeholders relevant to the MRC and that more emphasis on case studies that demonstrate how knowledge is generated would be of broad interest. The co-produced nature of the work meant the results could immediately inform the ongoing work to prepare ICS material.

The new typology of ICS attributes created during this work can be used to determine MRC stakeholder preferences for ICS content, language, and presentation style. There was evidence that readers had an emotional response to the ICS narrative and often rated ICS presentation and the type of language more influential than a description of the research finding or significance of the impact. This work showed that if stakeholders are aware of a typology of attributes, it could change their perception of an ICS, indicating that there may be some use in familiarising stakeholders with the typology of attributes used in this study before any formal evaluation of research outcomes.

It was found that the use of the term ICS promotes different expectations to readers than press releases, with the emphasis on using evidence and factual writing to provide a clear link between the research findings and wider societal benefits. Likewise, if ICS attempt to communicate challenges and issues around achieving impact from research, they may be more credible and useful to their intended audience.

## Supplementary Information

Supplementary Material 1.

Supplementary Material 2.

## Data Availability

All data generated or analysed during this study are provided within the manuscript or available from the corresponding author on reasonable request.

## Abbreviations

ICS: Impact Case Study
MRC: Medical Research Council
REF: Research Excellence Framework
UKRI: United Kingdom Research and Innovation
BBSRC: Biotechnology and Biological Sciences Research Council
STFC: Science and Technology Facilities council
EPSRC: Engineering and Physical Sciences Research Council
DSIT: Department for Science, Innovation and Technology
NHS: National Health Service
ANOVA: Analysis of Variance
ECR: Early Career Researcher
BBC: British Broadcasting Corporation
