# Supplementary material for: Identifying stakeholder preferences for communicating impact from medical research: a mixed methods study
Source: BMC Health Serv Res. 2024 Oct 29;24:1305. doi: 10.1186/s12913-024-11664-y (PMC11520885; doi:10.1186/s12913-024-11664-y)
Supplement: Supplementary file 1 — Supplementary Material 1. [file 12913_2024_11664_MOESM1_ESM.docx]

# Additional Information - Appendix

The appendix contains information supplementary to the main manuscript. Table 1A details a list of key attributes used during the semi-structured interviews. Table 2A details the four sample impact case studies used during the semi-structured interviews, the images are publicly available and used under the creative commons licence. Table 2B details the attributes present in the impact case studies. Table 3A details a list of the different types of impact used in the online survey when asking participants what type of impact they expected from medical research. Table 4A details the additional attributes suggested by participants at the end of the semi-structured interviews. Figure 5A shows a word cloud created from participant’s descriptions of impact from research. Appendix 6 details supporting quotes that were not included in the manuscript.

# Appendix 1: List of Key Attributes

Table 1A: List of different content, language and presentation attributes found in ICS.

| **Attribute** |
| --- |
| **Content** |
| Description of research discovery |
| Statement of problem (with quantitative indicators) |
| Attribution to the researchers |
| Attribution to the institution |
| Attribution to the funding scheme |
| Mention of collaborations and partnerships (public/private partnerships/patient involvement) |
| Clear link from research to impacts |
| Description of specific type of impact (economic/health/societal) |
| Description of economic impact |
| Description of health impact |
| Description of societal impact |
| Quote from research team (funders involved, other stakeholders involved in work) |
| Quote from outside research team (patient quote, external expert) |
| Suggestions for future work |
| **Language** |
| Reading age (9+?) |
| Academic language |
| Clear and direct description of actual impact, (e.g. no use of word “potential”) |
| Explanation of scientific breakthrough in lay language |
| Demonstrates causality between research and impact |
| Quantitative measures of evidence and impact |
| **Presentation** |
| Use of headings, strap lines |
| Images |
| Defined sections |
| Links to further info |
| Use of bullets and lists for information |

# Appendix 2: Impact Case Studies Chosen for Sample Pack

Table 2A – Example Impact Case Studies that formed the sample pack.

| ICS Number | Impact Case Study Title | Brief Description |
| --- | --- | --- |
| #1 | Motion Capture | UKRI website news story describing results from publications in Nature Medicine journal, highlighting progress made to predict disease progression using artificial technology and motion capture technology. |
| #2 | Avatar | BBC news story highlighting same motion capture technology project as #1 |
| #3 | Friendship Bench | University website impact case study describing introduction and global roll out of low-cost mental health intervention first trialed to treat depression in Southern Africa. |
| #4 | Gene Therapy | UKRI website impact case study detailing over 20 years of advancements in gene therapy aimed at curing rare diseases. |

**Case Study Example 1 Motion capture tech could predict disease progression**


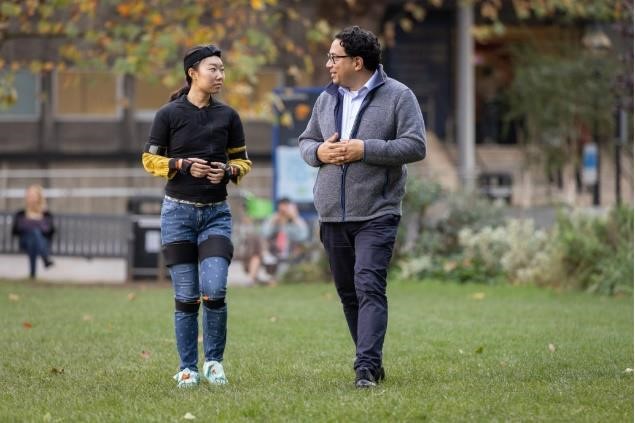


Credit: Imperial College London

A new wearable artificial intelligence (AI) technology could help to monitor the progression of movement disorders.

In 2 studies, published in Nature Medicine, researchers demonstrate the potential of this new approach to increase the efficiency of clinical trials in Duchenne muscular dystrophy and Friedreich’s ataxia.

The team are combining human movement data gathered from wearable tech, similar to the motion capture approach used in films, with AI to:

- identify clear movement patterns
- predict future disease progression

**Diagnosing disease**

They hope that it could also one day be used to monitor or diagnose a range of common diseases that affect movement behaviour such as:

- dementia
- stroke
- orthopaedic conditions

The research was funded by UK Research and Innovation (UKRI) through:

- a Turing AI Fellowship
- UKRI Centre for Doctoral Training (CDT) in AI for Healthcare
- Medical Research Council London Institute of Medical Sciences (MRC LMS)

The UKRI CDT in AI for Healthcare and AI Turing Fellowships are delivered for UKRI by the Engineering and Physical Sciences Research Council (EPSRC).

**Unprecedented, precise predictions**

Senior and corresponding author of both papers, UKRI Turing AI Fellow Professor Aldo Faisal from Imperial College London, said:

“Our approach gathers huge amounts of data from a person’s full-body movement, more than any neurologist will have the precision or time to observe in a patient. Our AI technology builds a digital twin of the patient and allows us to make unprecedented, precise predictions of how an individual patient’s disease will progress. We believe that the same AI technology working in 2 very different diseases, shows how promising it is to be applied to many diseases and help us to develop treatments for many more diseases even faster, cheaper and more precisely.”

**Improving diagnosis and monitoring**

Co-author of both studies Professor Richard Festenstein, from the MRC LMS and Department of Brain Sciences at Imperial said:

“Patients and families often want to know how their disease is progressing, and motion capture technology combined with AI could help to provide this information. We’re hoping that this research has the potential to transform clinical trials in rare movement disorders, as well as improve diagnosis and monitoring for patients above human performance levels. “ Partners in the research also include:

- UCL Great Ormond Street Institute for Global Health
- National Institute for Health and Care Research (NIHR) Great Ormond Street Hospital Biomedical Research Centre
- Ataxia Centre at UCL Queen Square Institute of Neurology
- Great Ormond Street Hospital
- National Hospital for Neurology and Neurosurgery
- University of Bayreuth
- Gemelli Hospital in Rome
- NIHR Imperial College Biomedical Research Centre

[**Link to Imperial College London website**](https://www.imperial.ac.uk/news/242711/wearable-tech-ai-clinical-teams-join/)

[**Link to BBC News story**](https://www.bbc.co.uk/news/science-environment-64326125)

**Case Study Example 2**

[**Motion capture tech from Avatar films used in disease research**](https://www.bbc.co.uk/news/science-environment-64326125)


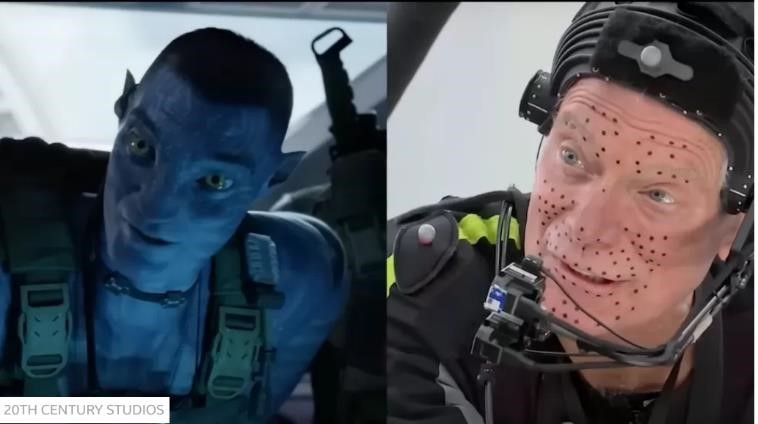


The Avatar films used sensors to capture the movement of actors to make them look like aliens. Scientists have adapted the technology to track the progression of diseases

Credit: 20^th^ Century Studios

**Motion capture suits that bring characters to life in films like Avatar are helping researchers track the onset of diseases which impair movement.**

The new system uses artificial intelligence to analyse body movements.

In tests, the UK experts measured the severity of two genetic disorders twice as quickly as the best doctors.

The researchers say it could also halve the time and greatly reduce the cost required to develop new drugs in clinical trials.

Dr Valeria Ricotti, of Great Ormond Street Institute for Child Health was among a group of researchers at Imperial College and University College London who spent 10 years developing the new technology. They tested it on patients with Friedreich's ataxia (FA) and Duchenne Muscular Dystrophy (DMD) in two separate studies.

The researchers say it could also be used to monitor patients recovering from other diseases that affect movement. These include any condition involving the brain and nervous system, heart, lungs, muscles, bone and a number of psychiatric disorders.

Prof Aldo Faisal of Imperial College, who was one of the scientists who came up with the idea, said it was an enormous improvement. "Our new approach detects subtle movements that humans can't pick up on," he said. "It has the capability to transform clinical trials as well as improve diagnosis and monitoring for patients."

FA typically appears in adolescence and affects one in 50,000 people, whereas DMD affects 20,000 children, mostly boys, globally each year. There is currently no cure for either.

A team at Imperial College first tested the motion sensor suits on patients with FA. They found that the AI could predict the worsening of the disease over twelve months, half the time it would normally take an expert.

A separate team at Great Ormond Street tested the technology on 21 boys with DMD between the ages of five and 18. It predicted how their movement would be affected six months in the future much more accurately than a doctor.

The researchers believe that their system could be used to speed up and lower the cost of clinical trials to test out new drugs for a wide range of conditions. In particular, it may make trials of new drugs for rare genetic disorders more cost effective.

Professor Paola Giunti, Head of UCL's Ataxia Centre said: "We will be able to trial more drugs with less patients at a lower cost."

In the case of DMD a minimum of 100 patients are needed over the course of about 18 months to get statistically significant results relating to the effectiveness of a new drug. The study showed that using the new system it could potentially be done with 15 patients over six months.

About 6,000 rare genetic diseases affect a total of about 1 in 17 people in the UK. The number of patients with each disease can amount to just a few hundred or less. That is a disincentive for drug companies to undertake expensive clinical trials to develop new medicines to treat them.

**Game Changer**

Professor Richard Festenstein from the Medical Research Council’s London Institute of Medical Sciences, told BBC News that the suit technology, which he helped to develop, had the potential to change the economics of drug discovery.

''This is going to attract the pharmaceutical industry to invest in rare diseases," he said. "The main beneficiary from our research is going to be patients, because the technology is going to be able to come up with new treatments much more quickly.''

The researchers are already seeking approval for the use of motion capture for drug trials for FA and DMD, which if successful could begin in two years. They are also gathering data for its use with Parkinson's, Alzheimer's and MS.

More on this story [AI system may diagnose dementia in a day](https://www.bbc.co.uk/news/health-57934589)

**Case Study Example 3** **The Friendship Bench**


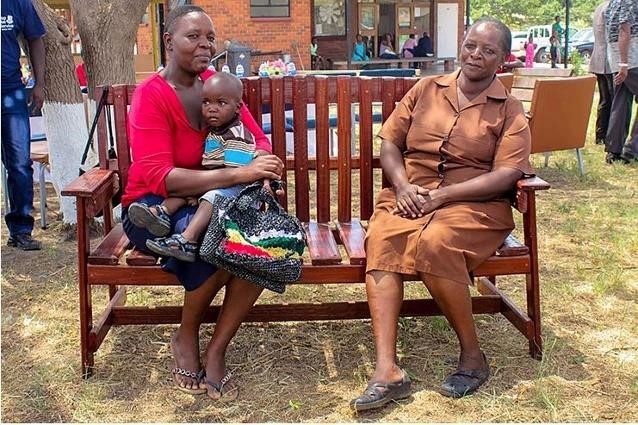


Credit: Centre for Global Mental Health

The Friendship Bench (FB) project is an evidence-based intervention developed in Zimbabwe to bridge the mental health treatment gap. The FB aims to enhance mental well-being and improve quality of life through the use of problem-solving therapy delivered by trained lay health workers, focussing on people who are suffering from common mental disorders, such as anxiety and depression.

The Friendship Bench intervention has been developed over a twenty-year period from community research in Zimbabwe. It uses a cognitive behavioural therapy-based approach at primary care level to address 'kufungisisa' – the local word closest to depression (literally, “thinking too much” in Shona).

Uniquely, the FB uses ‘grandmothers’ to deliver the therapy. These grandmothers are community volunteers, without any prior medical or mental health experience, who are trained to counsel patients usually for six structured 45-minute sessions, on wooden benches within the grounds of clinics in a discrete area.

**Impact**

In 2016, the results from a FB randomized controlled trial were published in JAMA, showing that the group from the Friendship Bench had a significant decrease in depressive symptoms, compared to the control group.

Since 2006, Dr Chibanda and his team have trained over 600 of the grandmothers in evidencebased talk therapy, which they deliver for free in more than 70 communities in Zimbabwe, and in 2017 alone 30,000 were seen on a Friendship Bench. The FB has now expanded beyond Zimbabwe; it is being used in Malawi and Zanzibar, and it has been adapted for New York City, highlighting that interventions created in low- and middle-income countries can be adapted for highincome countries. There are several FB studies currently underway including; The Youth Friendship Bench (YouFB), OptFB, FB Plus and Zvandiri.

**Project Team**

Dr Dixon Chibanda (Principal Investigator) London School of Hygiene & Tropical Medicine and University of Zimbabwe

Prof Melanie Abas

Prof Helen Weiss

Prof Ricardo Araya

Dr Vicky Simms

Dr Lorna Gibson

Prof Martin Prince

Dr Souci Frissa

Dr Helen Jack

Dr Ruth Verhey

Dr Bradley Wagenaar

**Funders**

Wellcome Trust, Grand Challenges Canada, NIHR, Comic Relief, GACD, MRC, CIFF, ZHTS

**Case Study Example 4 MRC funded discovery science underpins gene therapy cures**


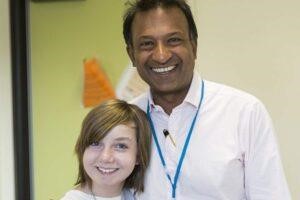


Professor Bobby Gaspar and Teigan, who received treatment for severe combined immunodeficiency.

Credit: Great Ormond Street Hospital

Revolutionary gene therapy technique, developed with support from MRC, has led to life-saving treatment for rare genetic childhood disease.

Metachromatic leukodystrophy (MLD) is a rare genetic disease that effects children and causes severe damage to the nervous system and organs, resulting in a life expectancy of between just 5 and 8 years.

In February 2023, [**it was announced by the NHS**](https://mft.nhs.uk/2023/02/15/first-baby-receives-life-saving-gene-therapy-on-nhs-at-royal-manchester-childrens-hospital/) that a 19-month-old baby had become the first child in the UK to receive a life-saving gene therapy treatment for MLD.

Previously, it was not possible to stop the disease and treatment was aimed at relieving symptoms using a variety of drugs to ease muscle spasms, treat infections and control seizures.

**Metachromatic leukodystrophy**

MLD is caused by the abnormal function of an enzyme that breaks down unwanted substances, called sulphatides, in the brain.

Curing the disease requires adding in a good version of the gene for the enzyme by a 1-time therapy called ‘Libmeldy’. The therapy works by taking stem cells from the patient through a bone marrow extract and then genetically correcting the cells outside of the body by replacing the faulty gene. These corrected cells are then transplanted back into the body, through an intravenous infusion. Sometimes the patient is given mild chemotherapy before the transplant; this is done to eliminate any existing stem cells that carry the genetic defect so that the new engineered cells can repopulate the bone marrow more effectively after the transplant.

**Gene therapy using lentiviral vectors**

The development of gene therapy for inherited childhood diseases such as MLD has required long term research funding investment.

The Medical Research Council (MRC) has been a major funder of UK gene therapy research for more than 20 years. This includes Professor Gaspar’s studies of rare inherited childhood diseases and lentiviral vectors that have formed the basis of this MLD breakthrough.

**‘Bubble boy disease’**

One of Professor Gaspar’s early successes was the development of a treatment of the rare immune disorder ‘bubble boy disease’.

‘Bubble boy disease’ is so called because affected children have severe combined

immunodeficiency (SCID) and are extremely vulnerable to infectious diseases, some of them had become famous for living in a sterile environment.

In the most severe forms, children with SCID are unable to fight off even very mild infections and, without treatment, will usually die within the first year of life.

Several years of research was done by Bobby Gaspar at Great Ormond Street Hospital and the UCL Institute of Child Health. This focused on developing a gene therapy treatment for a type of SCID known as adenosine deaminase deficiency (ADA), characterised by the lack of an enzyme called adenosine deaminase.

Support from MRC’s Developmental Pathway Funding Scheme took this therapy, now called OTL101, into the clinic and supported the establishment of Orchard Therapeutics.

**Orchard Therapeutics**

In 2017, both US and UK drug regulatory authorities granted OTL-101 designations reserved for treatments addressing high unmet need. These developments showed the commercial potential of Professor Gaspar’s work and highlight gene therapy’s ability to improve human health.

In April 2018, GlaxoSmithKline signed a strategic agreement to transfer its rare disease gene therapy portfolio to Orchard Therapeutics, strengthening Orchard’s position as a global leader in gene therapy for rare diseases.

In May 2021 the researchers followed up 50 patients treated for ADA-SCID with OTL-101, and the results showed 100% survival. Over 95% of the patients had sustained expression of the ADA enzyme showing that the gene therapy was successful, after 2 to 3 years following the treatment.

Table 2B – Key attributes contained in the impact case study examples, (green indicates attribute present, red indicates attribute absent in an impact case study).

| **Attribute** | **ICS 1** | **ICS 2** | **ICS 3** | **ICS 4** |
| --- | --- | --- | --- | --- |
| **Content** |  |  |  |  |
| Description of research discovery |  |  |  |  |
| Statement of problem (with quantitative indicators) |  |  |  |  |
| Attribution to the researchers |  |  |  |  |
| Attribution to the institution |  |  |  |  |
| Attribution to the funding scheme |  |  |  |  |
| Mention of collaborations and partnerships (public/private partnerships/patient involvement) |  |  |  |  |
| Clear link from research to impacts |  |  |  |  |
| Description of specific type of impact (economic/health/societal) |  |  |  |  |
| Description of economic impact |  |  |  |  |
| Description of health impact |  |  |  |  |
| Description of societal impact |  |  |  |  |
| Quote from research team (funders involved, other stakeholders involved in work) |  |  |  |  |
| Quote from outside research team (patient quote, external expert) |  |  |  |  |
| Suggestions for future work |  |  |  |  |
| **Language** |  |  |  |  |
| Reading age (9+?) |  |  |  |  |
| Academic language |  |  |  |  |
| Clear and direct description of actual impact, (e.g. no use of word “potential”) |  |  |  |  |
| Explanation of scientific breakthrough in lay language |  |  |  |  |
| Demonstrates causality between research and impact |  |  |  |  |
| Quantitative measures of evidence and impact |  |  |  |  |
| **Presentation** |  |  |  |  |
| Use of headings, strap lines |  |  |  |  |
| Images |  |  |  |  |
| Defined sections |  |  |  |  |
| Links to further info |  |  |  |  |
| Use of bullets and lists for information |  |  |  |  |

# Appendix 3: List of Expected Types of Impact from Online Qualtrics Survey

Table 3A: List of Different Types of Impact

| **Type of Impact** |
| --- |
| Improved health and wellbeing |
| High number of publications and citations |
| Impacts on understanding, learning and participation |
| Impacts on public policy, law, and services |
| Impacts on practitioners and practices |
| Economic benefits |
| Improvements in social welfare |
| Impacts on creativity, culture, and society |
| Environmental benefits |
| Manufacturing improvements |

# Appendix 4: List of additional attributes from MRC Stakeholders

At the end of the semi-structured interviews stakeholders were asked for suggestions of additional attributes they would like to see in an ICS. Table 4A shows the results.

Table 4A: List of Attributes suggested by MRC stakeholders to include in an impact case study.

| **Attribute** | **Attribute Type** | **Suggested by** |
| --- | --- | --- |
| Content relevant to the reader (area of expertise) | Content | NHS |
| Personal interest/emotional connection | Language | Industry |
| Writing must be persuasive | Language | Academia – Senior Management |
| Engaging and grabs attention | Language | Industry / Government / NHS |
| Short and concise | Language | Medical Research Charity / Academia |
| Have a concise summary with option to read more | Language | Medical Research Charity |
| A planned timetable for future work | Content | Industry |
| Highlighting barriers or opportunities | Content | Industry |
| Scope for further potential impact | Language | Industry |
| What could not be achieved, and what was learned from that. | Content | Academia – Senior Management |
| Clear title | Presentation | Academia - ECR |
| Clear description of beneficiaries | Content | Academia |
| Quote from the person who has been impacted – quote from a real person whose life was made better by the research | Content | Industry |
| Visual summary – like a video/artistic depiction/graphical abstract | Presentation | Academia - ECR |
| More context, how has it moved the field on, more powerful statements into why the research is world leading/transformational | Language | Medical Research Charity |
| Sustainability of the impact, and ethics of the impact. | Content | Academia – Senior Management |
| What were the unexpected/unintended impact. | Content | Academia – Senior Management |
| Need statistical information on scale of problem, cost spent on research and scale of impact to assess the value of a case. | Content | Government |
| Economic impact, especially impact for the UK | Content | Government |

Over half of the additional attributes suggested by the stakeholders were content, over a third were language and just over 10 percent were presentation attributes.

# Appendix 5: Word cloud of keywords describing impact


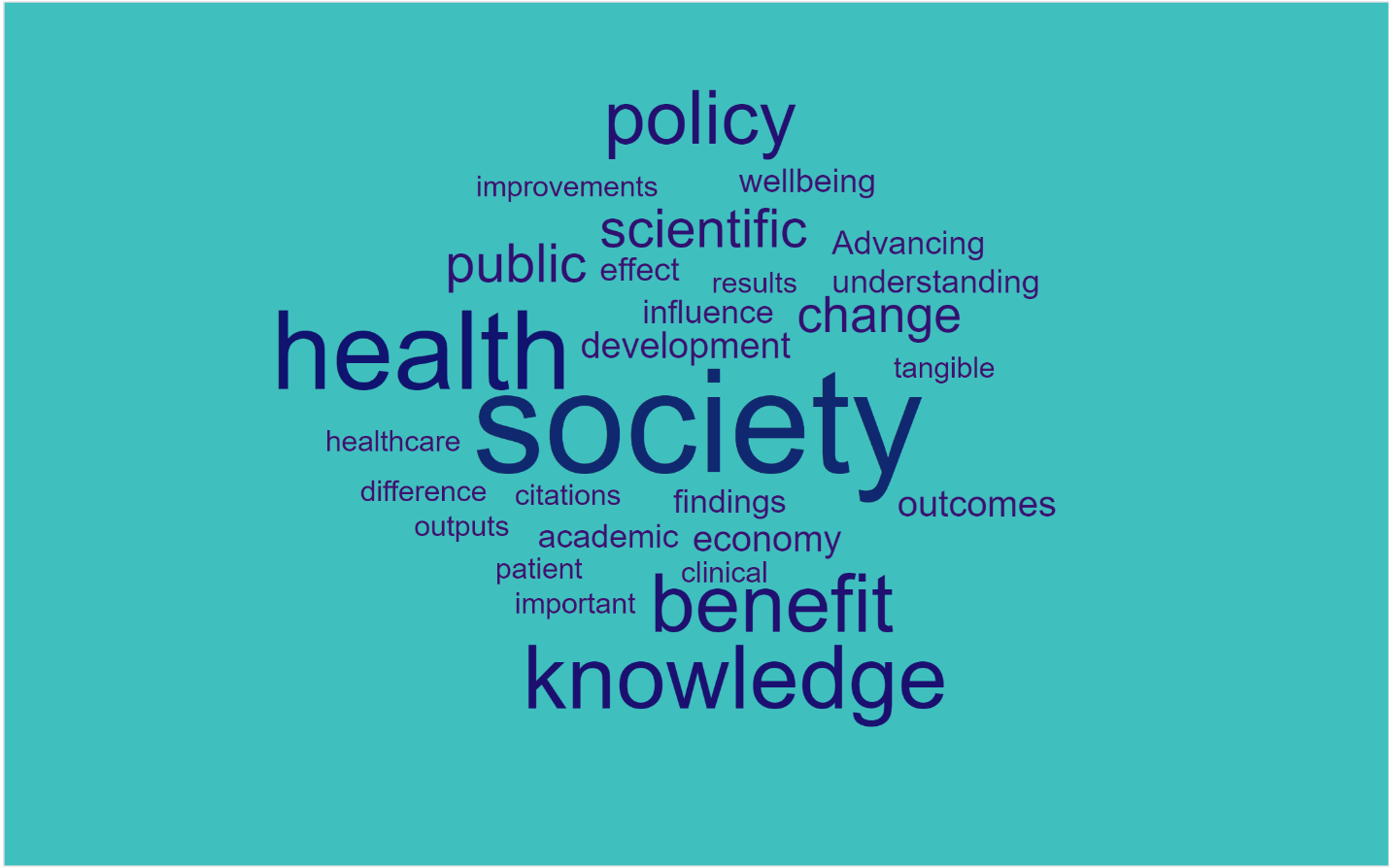


Figure 5A: Word cloud created from MRC stakeholders' descriptions of impact from research. More frequently occurring words are shown in larger font.

# Appendix 6: Quotes from Stakeholders that support key topics

Topic: When writing impact narratives consideration of the intended audience is key and content should be tailored to these audiences.

***“I quite like the bit setting out the commercial benefits, so setting out the deal with GSK and where it is with the*** ***FDA.”***

*MRC Stakeholder from Government about #4*

Topic: Care should be taken to include factual evidence to clearly describe the actual impact and what might be achieved in the future.

***“******it's very sensationalised, a lot of the communication*** ***that's made around science. If you go to a newspaper, they obviously have an agenda.”***

*MRC Stakeholder from NHS*

Topic: Specific presentation and language attributes provide emotional aspects to the narrative and can determine preferences more than content attributes.

***“I think*** ***it's*** ***really important that it be evidence based and because, you know, we all do the hype. You know, if you have a piece of research and you want to catch a press and media attention, you can put some more hype on it.”***

*MRC Stakeholder from Academia – Senior Management*

***“******game changer...possibly I'm being dramatic and excessive, but maybe it was a bit of hyperbole”.***

*MRC Stakeholder from Academia – ECR*

***“******It's important, in the sense, the difference between news, research news and an impact case study*** ***is that there needs to be some academic message, so academic language would be useful. That will give the professional articulation so that it looks like*** ***it's a serious topic.”***

MRC Stakeholder from Academia – Early Career Researcher (ECR)

On use of bullets and lists

***“It is very structured; the bullet points help you understand the key facts and messages.”***

MRC Stakeholder from Industry

***“I think*** ***that's quite disengaging for people, just lists of things. People tend to just not read them. You lose the story when*** ***you're listing things for people.”***

MRC Stakeholder from Medical Research Charity

On use of catchy titles

***“Catchy titles can be a bit counterproductive; I strongly agree that the title is fundamental… but not catchy here.”***

MRC Stakeholder from Academia

On use of quotes

***“I think that's people getting their name on stuff because they've been asked to……. it’s free publicity for you as an expert.”***

*MRC Stakeholder from Industry*
