# Supplementary material for: Identifying stakeholder preferences for communicating impact from medical research: a mixed methods study
Source: BMC Health Serv Res. 2024 Oct 29;24:1305. doi: 10.1186/s12913-024-11664-y (PMC11520885; doi:10.1186/s12913-024-11664-y)
Supplement: Supplementary file 2 — Supplementary Material 2. [file 12913_2024_11664_MOESM2_ESM.pdf]

MRC Impact Case Studies

Start of Block: Block 3

MRC Impact Case Study Questionnaire

Many thanks for answering a few quick questions about the benefits of research, this survey should take approximately 5-10 min to complete.

You will be asked for your consent to take part in this research project below.

This survey will gather participants attitudes and opinions about the use of impact case studies to communicate outcomes from research funded by the Medical Research Council. You will be asked to leave an email address if you wish to take part in further research for this project. The results will be used to develop future impact case studies for internal and external communications.

If you have any questions from the above information, please contact the researcher, Katherine.Pitrolino@ukri.org, before you decide whether to join in. Upon completing the study, you have 2 weeks to withdraw from the project by contacting the researcher above.

I agree that:

• I have read the notes written above and understand what the study involves. I have had the opportunity to ask questions and these have been answered satisfactorily.

• I understand that if I decide at any time that I no longer wish to take part in this project, I can notify the researchers involved within 2 weeks and withdraw immediately.

• I understand that I can leave my email address if I wish to be contacted and take part in further research.

• I consent to my questionnaires and written feedback (including anonymised quotes) being used for the purposes of this research study. I understand that my information can be accessed by members of the research team. I give permission for these individuals to have access to my research notes for this study.

• I understand that my data will be treated as strictly confidential and handled in accordance with the provisions of the Data Protection Act 1998.

• I understand that the information I have submitted will be published as a report and may contribute to a publication.

• I agree that the research project named above has been explained to me to my satisfaction and I agree to take part in this study.

○ I consent (1)

○ I do not consent (2)

End of Block: Block 3

Start of Block: Default Question Block

Which of the following describes your work sector or organisation? Please choose all that apply.

- ☐ Academy / Society (4)
- ☐ Medical Research Charity (5)
- ☐ University - Senior Management (6)
- ☐ University - Early Career Researcher (7)
- ☐ Research Centre / Institute (8)
- ☐ Healthcare Sector (9)
- ☐ Regional Consortia (10)
- ☐ Trade Associations (11)
- ☐ MRC funding panel member (12)
- ☐ Other (19) ____

How would you describe impact from research?

____

Page Break

What kind of impacts do you expect from MRC research?
Please rate the following types of impact on a scale of 1-10, where 1 means not expected from MRC research, 10 being fully expected from MRC research.

	0 1 2 3 4 5 6 7 8 9 10
High number of publications and citations ()	
Improved health and wellbeing ()	
Impacts on creativity, culture and society ()	
Improvements in social welfare ()	
Economic benefits ()	
Impacts on public policy, law and services ()	
Manufacturing improvements ()	
Impacts on practitioners and delivery of professional services, enhanced performance or ethical practice ()	
Environmental benefits ()	
Impacts on understanding, learning and participation ()	

Are these expectations currently being met?

- ◯ Yes (1) ____________________
- ◯ Maybe (2) ____________________
- ◯ No (3) ____________________

How do you currently find out about these impacts?

____

Could anything more be done to improve the current content you receive?

____

End of Block: Default Question Block

Start of Block: Block 2

If you are you happy for us to contact you in the future for further information, please leave your email address below. Thank you.

____

End of Block: Block 2
